# Evolution and clustering of prodromal parkinsonian features in *GBA1* carriers

**DOI:** 10.1002/mds.27775

**Published:** 2019-06-28

**Authors:** Stephen Mullin, Michelle Beavan, Jonathan Bestwick, Alisdair McNeill, Christos Proukakis, Timothy Cox, Derralynn Hughes, Atul Mehta, Henrik Zetterberg, Anthony H.V. Schapira

**Affiliations:** ^1^ Department of Clinical and Movement Neurosciences University College London Queen Square Institute of Neurology London United Kingdom; ^2^ Institute of Translational and Stratified medicine Plymouth University Peninsular School of Medicine Plymouth United Kingdom; ^3^ Wolfson Institute of Preventive Medicine, Barts and The London School of Medicine and Dentistry Queen Mary University of London London United Kingdom; ^4^ Sheffield Institute of Translational Neuroscience University of Sheffield Cambridge United Kingdom; ^5^ Department of Medicine University of Cambridge Cambridge United Kingdom; ^6^ Lysosomal Storage Disorders Unit, Royal Free Hospital, Royal Free London NHS Foundation Trust, and Department of Haematology University College London London United Kingdom; ^7^ Department of Psychiatry and Neurochemistry the Sahlgrenska Academy at the University of Gothenburg Mölndal Sweden; ^8^ Department of Molecular Neuroscience University College London Institute of Neurology London United Kingdom

**Keywords:** cognition, depression, glucocerebrosidase, Gaucher, olfaction, Parkinson's, prodromal

## Abstract

**Background:**

Five to 25% of patients with PD carry glucocerebrosidase gene mutations, and 10% to 30% of glucocerebrosidase carriers will develop PD by age 80. Stratification of PD risk in glucocerebrosidase carriers provides an opportunity to target disease‐modifying therapies.

**Objective:**

Cross‐sectional and longitudinal survey of prodromal PD signs among glucocerebrosidase carriers.

**Design:**

Prospective assessment of 82 glucocerebrosidase mutation carriers and 35 controls over 4 to 5 years for prodromal clinical PD features.

**Results:**

At all time points, olfactory (measured using University of Pennsylvania Smell Identification Test) and cognitive (Montreal Cognitive Assessment) function and the International Parkinson and Movement Disorder Society UPDRS parts II and III scores were significantly worse amongst glucocerebrosidase mutation carriers. Progression to microsmia (odds ratio: 8.5; 95% confidence interval: 2.6–28.2; *P* < 0.05) and mild cognitive impairment (odds ratio: 4.2; 95% confidence interval: 1.1–16.6; *P* < 0.05) were more rapid compared to controls. Those with worse olfaction also had worse cognition (OR, 1.5; 95% CI: 0.0–2.8; *P* < 0.05) and depression (OR, 1.3; 95% CI: 0.6–2.8; *P* < 0.05). No participants reached the MDS prodromal PD diagnostic criteria before PD diagnosis. One participant developed PD. He did not fulfill the International Parkinson and Movement Disorder Society prodromal PD criteria before diagnosis.

**Conclusion:**

Assessment of individual and clustered PD prodromal features may serve as a useful tool to identify high‐risk subjects for conversion to PD. As a result of the low conversion rate in our glucocerebrosidase mutation carriers to date, prospective validation is needed in larger cohorts to establish the profile of these features in PD convertors. © 2019 The Authors. *Movement Disorders* published by Wiley Periodicals, Inc. on behalf of International Parkinson and Movement Disorder Society.

Mutations in the lysosomal glucocerebrosidase gene (*GBA1*) are the most significant genetic risk factor for Parkinson disease (PD). Five to 10% of PD patients carry a *GBA1* mutation, rising to 25% in high‐risk groups such as Ashkenazi Jews.^1^ In the biallelic (i.e., homozygous or compound heterozygous carrier of a *GBA1* mutation) state, *GBA1* mutations cause the lysosomal storage disorder, Gaucher disease (GD).^2^ Biallelic and heterozygous *GBA1* mutations increase the risk for PD and dementia with Lewy bodies,^1^, ^3^ but display incomplete penetrance for PD with 10% to 30% affected by age 80.^4^, ^5^ Those *GBA1* mutations associated with the more severe neuronopathic form of GD have a higher risk of developing PD.^6^ The two most common mutations are N370S and L444P. Respectively, they increase the risk of PD by ≈4‐ and ≈8‐ to 12‐fold.^7^ Common *GBA1* variants, such as T369M and E326K, increase the risk by ≈1.5‐fold, but are not associated with GD.^7^

The *GBA1* metabolic pathway is an attractive target for neuroprotective therapy in PD.^8^, ^9^ Disease‐modifying treatments are likely to be most effective when given early, for instance during a prodromal phase of microsmia, constipation, depression, dysautonomia, rapid eye movement sleep behavior disorder (RBD), and cognitive impairment. We present the third prospective assessment for prodromal PD features of a cohort of *GBA1* mutation carriers, 4 to 5 years after baseline.^10^, ^11^ Our findings suggest that a combination of clinical markers may help define a subgroup of *GBA1* mutation carriers at increased risk of PD.

## Materials and Methods

### Participants

Type 1 GD patients aged >40 years were recruited from the Royal Free London Hospital from 2010. These patients are biallelic for *GBA1* mutations. Heterozygous *GBA1* ^+^ relatives were recruited from their kindred. Controls were unrelated. The group of heterozygous *GBA1* carriers + biallelic carriers will be referred to as the combined *GBA1* group. All participants were free of neurological features at inclusion.^10^, ^11^ Participants were assessed longitudinally from baseline (2010–2011), with target assessments in 2012–2013 (time point 1) and 2014–2015 (time point 2). Previous studies referred to age‐matched populations, whereas the present analysis encompasses these and additional cases drawn from our longitudinal database. The study was approved by the Hampstead Research Ethics Committee (10/H0720/21). A full list of subjects’ mutations is available in Supporting Information Table S1.

**Figure 1:**
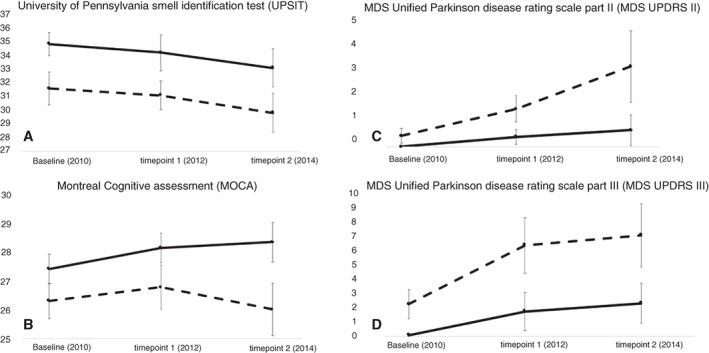
Trimmed means of (A) University of Pennsylvania smell identification test (UPSIT), (B) Montreal cognitive assessment (MOCA), and Movement disorders society unified Parkinson disease ratings scale (MDS UPDRS) part II (C) and III (D) scores of combined cohort of GBA carriers (dashed line) and controls (solid line) with exact confidence intervals. At baseline there were 117 participants (35 controls, 39 heterozygous and 43 bi‐allelic), 103 at time‐point 1 (28 controls, 33 heterozygous and 42 bi‐allelic) and 85 at time‐point 2 (19 controls, 26 heterozygous and 40 bi‐allelic).

We used a rolling recruitment model to enrol new participants throughout the duration of the study (see Supporting Information Fig. S2). These participants had their first assessment added to the baseline group regardless of the year of assessment. At baseline, there were 117 participants (35 controls, 39 heterozygous, and 43 biallelic), 103 at time point 1 (28 controls, 33 heterozygous, and 42 biallelic), and 85 at time point 2 (19 controls, 26 heterozygous, and 40 biallelic). In a rolling recruitment model, new participants were enrolled into the study in 2012–2013 (2 control, 1 heterozygous, and 2 biallelic) and 2014–2015 (3 control, 3 heterozygous, and 1 biallelic) and analyzed as baseline. Eleven control participants dropped out of the study (4 at time point 1 and 7 at time point 2; 31%), 3 declined to take part, 4 were lost to follow up, 2 were too frail/ill to undergo assessment, and 2 died. In the heterozygous group, there were 9 dropouts (23%; 3 at time point 1 and 6 and time point 2). Three declined to take part, 3 were lost to follow‐up, and 3 were too frail/ill to undergo assessment. In the bi‐allelic group, there were 2 dropouts (5%) both at time point 2. Both declined to take part. All participants lost to follow‐up were contacted by phone, e‐mail and post. A flow diagram of recruitment and retention is displayed in Supporting Information Figure S2.

### Follow‐up Evaluation

A standardized clinical history and neurological assessment was conducted, including the International Parkinson and Movement Disorder Society UPDRS (MDS‐UPDRS) parts II and III, the University of Pennsylvania Smell Identification Test (UPSIT), the Montreal Cognitive Assessment (MoCA), the Rapid Eye Movement Behaviour Disorder Sleep Questionnaire (RBDSQ), and the Beck's Depression Inventory (BDI). Participants were examined independently by a physician trained in movement disorders (A.M., M.B., or S.M.). A blinded senior movement disorders neurologist (A.H.V.S.) evaluated participants upon deterioration in MDS‐UPDRS scores.

### Genotyping

Sequencing of the *GBA1* gene was carried out as described previously^12^ (primers available on request). All subjects were genotyped, including controls.

### Standardization of MDS‐UPDRS Testing

To ensure consistency in MDS‐UPDRS part III scoring, investigators carried out a blinded assessment of 4 patients with a clinical diagnosis of PD and compared scores. Across the MDS‐UPDRS III section, average score deviation was 1.75 points (range, 0–4).

### Missing Data

A sensitivity analysis, in which a regression model for respective dependent variables at each time point, was constructed and showed data were “missing at random.” For each variable, percentages of missing data (including missing data for participants who dropped out of the study) were as follows:

**Table:** 

	Baseline	Time Point 1	Time Point 2
UPSIT	7%	21%	32%
MoCA	4%	20%	33%
RBDSQ	1%	25%	35%
BDI	30%	35%	32%
MDS‐UPDRS II	0%	20%	34%
MDS‐UPDRS II	1%	20%	33%

We used multiple imputation using chained equations (m = 50) to impute missing data. All study covariates were included in the derivation of imputed values.

### Clinical Data Analysis

Data were analyzed using Stata software (version 14.1; StataCorp LP, College Station, TX). Graphs were plotted using Stata and Microsoft Excel (v.15.17; Microsoft Corporation, Redmond, WA).

#### Analysis Strategy

Our primary analysis was the combined *GBA1* versus control groups. For significant associations, we carried out secondary analyses comparing biallelic/heterozygous groups to controls. We also carried out a separate analysis looking at the influence of the total number of severe mutations across two alleles among the entire *GBA1* mutation carrying cohort.

### Study Covariates

We assessed the confounding effect of study covariates and variation of group demographics with descriptive statistics and a Kruskal‐Wallis test (Table 1). Covariates were included if there was evidence of a significant difference in the characteristics of the participants across different groups at any time point (*P* < 0.05) and/or if there was a strong biological rationale for their inclusion. As a result, the following study covariates were included: sex, education (university/nonuniversity educated), family history of PD/dementia, number of severe *GBA1* mutations, significant past smoking history (>1 pack year), and age.

**Table 1:** Demographics of study groups

	Baseline n = 117	Time Point 1 n = 103	Time Point 2 n = 85
Control median age	61	59	59
Biallelic median age	51	52	52.5
Heterozygous median age	60	63	61.5
Significance level	*P* = 0.002	*P* = 0.017	*P* = 0.031
*GBA1* carriers median age	55	56	56
Significance level (vs control)	*P* = 0.142	*P* = 0.505	*P* = 0.748
Control % male (n)	47 (16)	57 (16)	56 (11)
Biallelic % male (n)	58 (24)	61 (24)	55 (22)
Heterozygous % male (n)	45 (18)	40 (13)	35 (9)
Significance level	*P* = 0.537	*P* = 0.282	*P* = 0.221
*GBA1* % male (n)	51 (42)	50 (37)	49 (31)
Significance level (vs. control)	*P* = 0.396	*P* = 0.404	*P* = 0.350
Control % family history PD/dementia (n)	6 (2)	8 (2)	11 (2)
Biallelic % family history PD/dementia (n)	23 (10)	21 (9)	22 (9)
Heterozygous % family history PD/dementia (n)	10 (4)	15 (5)	19 (5)
Significance level	*P* = 0.062	*P* = 0.308	*P* = 0.315
*GBA1* % family history PD/dementia (n)	17 (14)	17 (14)	22 (14)
Significance level (vs control)	*P* = 0.084	*P* = 0.253	*P* = 0.226
Control % university educated (n)	68 (24)	63 (17)	74 (14)
Biallelic % university educated (n)	48 (21)	49 (21)	45 (18)
Heterozygous % university educated (n)	50 (20)	53 (14)	50 (13)
Significance level (vs. control)	*P* = 0.247	*P* = 0.625	*P* = 0.100
*GBA1* % university educated (n)	0.49 (41)	0.50 (35)	0.48 (31)
Significance level (vs. control)	*P* = 0.075	*P* = 0.199	*P* = 0.030
Control % smoker (n)	31 (11)	33 (9)	42 (8)
Biallelic % smoker (n)	30 (13)	29 (12)	29 (12)
Heterozygous % smoker (n)	36 (14)	33 (11)	42 (11)
Significance level	*P* = 0.851	*P* = 0.904	*P* = 0.459
*GBA1* % smoker (n)	27 (27)	31 (23)	34 (33)
Significance level (vs. control)	*P* = 0.527	*P* = 0.510	*P* = 0.361

At baseline, there were 117 participants (35 controls, 39 heterozygous, and 43 biallelic), 103 at time point 1 (28 controls, 33 heterozygous, and 42 biallelic), and 85 at time point 2 (19 controls, 26 heterozygous, and 40 biallelic); 110 participants (32 controls, 36 heterozygous, and 42 biallelic).

### Sample Distributions

Non‐normally distributed data for all dependent variables precluded parametric analyses, taking into account covariate risk factors. None were amenable to transformation, so, where possible, we chose to stratify data using clinically validated thresholds.

### Cross‐Sectional Analyses

Where possible (MoCA, BDI, UPSIT, and RBDSQ), data were categorized according to clinically validated thresholds:

UPSIT: <19 anosmia, 20 to 24 severe microsmia, 25 to 29 microsmia, 30 to 33 mild microsmia^13^
MoCA: <25 dementia, 25 to 26 mild cognitive impairment^14^
RBDSQ: >5 RBD^15^
BDI: 9 to 19 mild depression, 20 to 29 moderate depression, >29 severe depression^16^

Ordinal logistic regression was used to determine associations between the combined *GBA1* cohort and controls, adjusted for study covariates (see above).

Mann‐Whitney U tests were used to compare dependent variables which were not amenable to stratification (MDS‐UPDRS II/III).

### Longitudinal Analyses

Repeated‐measures logistic regression was used to determine progression to mild cognitive impairment and microsmia, adjusted for study covariates. To carry out this analysis, it was necessary to dichotomize the data set:

UPSIT: <30 microsmia^13^
MoCA: <27 mild cognitive impairment^14^
RBDSQ: >5 RBD^15^
BDI: >8 mild depression^16^

A nonparametric trend analysis was undertaken in the case of significant findings to confirm that this was a true representation of change in scales. The nonparametric distribution and lack of a clinically validated threshold meant that we could not perform a longitudinal analyses for MDS‐UPDRS II and III.

### Trimmed Means

Outliers with disproportionately poor scores in *GBA1* carrier groups drove a non‐normal distribution. Trimmed means were derived by excluding participants with scores >2 standard deviations (SDs) from the mean (and recalculating). We did this to provide more representative descriptive statistics. Trimmed means did not form part of any of the statistical analyses.

### Severe Mutations

Within the combined *GBA1* group, we generated a logistic regression model assessing the influence (including all study covariates) of a number the severe mutations carried across two alleles (no severe mutations = 0, one severe mutations = 1, or two mutations = 2) on all dependent variables, together with a subset analysis within the heterozygous or biallelic group alone.

### Risk Stratification Procedure

To generate an indicator of overall worse/deterioration of performance, we devised a novel marker of prodromal PD progression and severity. From the first to last time point, an 0.5‐SD fall in the assessment score of each prodromal feature scored 1 point (maximum 4). Another point was scored for every 0.5 SD that the prodromal feature score of each participant was below the trimmed mean of the combined *GBA1* group (maximum 4). For each prodromal feature, this produced a combined risk score out of 8. A summary and example of the stratification system are displayed in Supporting Information Figure S1.

### Symptoms Clustering Analysis

To search for clustering of prodromal PD features in our *GBA1* group, we used our prodromal feature risk scores (see Risk Stratification Procedure section above) to generate a backward selection ordinal regression model examining potential associations between the UPSIT risk score (dependent variable) and other risk scores. The analysis included all study covariates. The final model was repeated in heterozygous/biallelic carriers/control groups.

### Application of MDS Diagnostic Criteria for Prodromal PD to Cohort

We applied the MDS diagnostic criteria for prodromal PD to our cohort. This produced a posttest probability percentage of developing PD for each patient. This was based upon the following data: age, smoking status, sex, history of constipation, symptomatic hypotension, erectile dysfunction, urinary dysfunction, family history of PD, presence of RBD (as defined by screening questionnaire), presence of depression, an MDS‐UPDRS III score > 3 (excluding action tremor), or the presence of a mild or severe *GBA1* mutation. Dopaminergic PET/single‐photon emission computed tomography imaging or data on excessive daytime somnolence were not available.

### Serum Alpha‐Synuclein Concentration

Serum was collected from 53 members of our longitudinal cohort (28 biallelic, 13 heterozygous, and 12 controls). This was supplemented by an additional 11 biallelic, 4 heterozygous, and 15 control serum samples. Median ages were: control 64, biallelic 52, and heterozygotes 60. Sixty‐five percent of the control group, 52% of the biallelic group, and 61% of the heterozygous group were male.

Serum alpha‐synuclein (A‐SYN) was measured using the Covance assay (Covance, Dedham, MA). All measurements were performed in duplicate in one batch by board‐certified laboratory technicians using the same reagents. A quality‐control sample was run at the beginning and end of each run. Technicians were blinded to clinical/genetic data. The limit of detection was 1.5 pg/mL.

To compensate for limits of sensitivity, A‐SYN concentrations were grouped in 10 equal strata (and scored 1–10). Ordinal logistic regression analysis was used to seek any association between these scores and *GBA1* status with age and sex as covariates (other demographics unavailable). We repeated the analysis using the combined UPSIT + MoCA + BDI risk score. We also carried out a post‐hoc analysis looking for an association between A‐SYN levels and the number of severe mutations carried.

## Results

### Cohort Characteristics

Group demographics are displayed in Table 1. A total of 117 subjects were included in the cross‐sectional analysis (35 controls, 39 heterozygous, and 43 biallelic), One hundred ten participants (32 controls, 36 heterozygous, and 42 biallelic) were included in the longitudinal analysis. Among the heterozygous group 28 (72%) had no severe mutations, and 11 (18%) had one severe mutation. Among the biallelic group, 11 (26%) had no severe mutations, 27 (63%) had one severe mutation, and 5 (11%) had two severe mutations. A full list of the mutations of the *GBA1*‐positive participants are shown in Supporting Information Table S1.

### Clinical Assessment Findings

At all three time points for *GBA1* mutation carriers, olfactory disturbance was more prevalent both within the combined GBA and heterozygous and biallelic groups (Table 2; Fig. 1). Progression to microsmia was faster among the combined *GBA1* group (odds ratio [OR]: 8.5; 95% confidence interval [CI]: 2.6–28.2; *P* < 0.05) and among the heterozygous (OR, 9.9; 95% CI: 2.7–35.9; *P* < 0.05) and biallelic (OR, 6.8; 95% CI: 1.6–28.1; *P* < 0.05) groups compared to controls (Fig. 1).

**Table 2:** Summary table of results

	Cross‐Sectional Analyses Combined *GBA1* ^*+*^ */Heterozygous/Biallelic Group* Versus Control						Longitudinal Analysis Combined *GBA1*/Heterozygous/Biallelic *Group* Versus Control (Repeated‐Measures Logistic Regression)	
	Baseline (2010–2011)		Time Point 1 (2012–2013)		Time Point 2 (2014–2015)			
University of Pennsylvania smell identification test (UPSIT): logistic regression/repeated measures logistic regression								
	Combined *GBA1* ^*+*^		Combined *GBA1* ^*+*^		Combined *GBA1* ^*+*^		Combined *GBA1* ^*+*^	
OR [95% CI]	7.4 [2.4–25.7]		7.9 [2.6–24.4]		6.3 [1.9–21.1]		8.5 [2.6–28.2]	
coef [95% CI]	2.1 [0.9–2.3] *P* < 0.05		1.9 [0.9–3.2] *P* < 0.05		1.8 [0.6–3.0] *P* < 0.05		2.1 [0.9–3.3] *P* < 0.05	
	Heterozygous	Biallelic	Heterozygous	Biallelic	Heterozygous	Biallelic	Heterozygous	Biallelic
OR [95% CI]	9.4 [2.4–35.4]	7.0 [2.0–24.8]	10.0 [2.8–36.1]	6.7 [2.0–22.4]	6.9 [1.8–26.7]	5.7 [1.5–21.7]	9.9 [2.7–35.9]	6.8 [1.6–28.1]
coef [95% CI]	2.2 [0.9–3.6]	1.9 [0.7–3.2]	1.9 [0.7–3.1]	2.3 [1.0–3.6]	1.9 [0.6–3.3]	1.7 [0.4–3.1]	2.3 [1.0–3.6]	1.9 [0.5–3.3]
	*P* < 0.05	*P* < 0.05	*P* < 0.05	*P* < 0.05	*P* < 0.05	*P* < 0.05	*P* < 0.05	*P* < 0.05
Montreal cognitive assessment (MoCA): logistic regression/repeated measures logistic regression								
	Combined *GBA1* ^*+*^		Combined *GBA1* ^*+*^		Combined *GBA1* ^*+*^		Combined *GBA1* ^*+*^	
OR [95% CI]	4.3 [1.5–12.5]		2.8 [1.2–9.0]		3.1 [1.1–10.4]		4.2 [1.1–16.6]	
coef [95% CI]	1.5 [0.4–2.5]		1.0 [0.1–2.2]		1.1 [0.1–2.3]		1.4 [0.1–2.8)	
	*P* < 0.05		*P* < 0.05		*P* < 0.05		*P* < 0.05	
	Heterozygous	Biallelic	Heterozygous	Biallelic	Heterozygous	Biallelic	Heterozygous	Biallelic
OR [95% CI] coef [95% CI]	5.4 [1.4–15.7] 1.6 [0.4–2.8] *P* < 0.05	3.9 [1.2–12.7] 1.4 [0.2–2.5] *P* < 0.05	Not significant	Not significant	Not significant	4.6 [1.2–17.8] 1.6 [0.2–2.9] *P* < 0.05	2.6 [1.6–11.4] 0.9 [0.5–2.4] *P* < 0.05	8.2 [1.6–41.0] 2.1 [0.5–3.7] *P* < 0.05
Beck's depression index (BDI): logistic regression/repeated measures logistic regression								
	Combined *GBA1* ^*+*^		Combined *GBA1* ^*+*^		Combined *GBA1* ^*+*^		Combined *GBA1* ^*+*^	
OR [95% CI]					10.6 [1.3–90.0]			
coef [95% CI]	Not significant		Not significant		2.2 [–0.1 to 4.3]		Not significant	
					*P* < 0.05			
OR [95% CI]					Heterozygous	Biallelic		
coef [95% CI]					17.4 [1.9–159.6] 2.6 [0.4–4.6]	ns		
					*P* = < 0.05			
Rapid eye movement disorder sleep questionnaire (RBDSQ): logistic regression/repeated measures logistic regression								
	Not significant		Not significant		Not significant		Not significant	
MDS‐UPDRS Part II: Kruskal‐Wallis								
	Combined *GBA1* ^*+*^		Combined *GBA1* ^*+*^		Combined *GBA1* ^*+*^			
	Chi^2^ = 6.1		Chi^2^ = 12.5		Chi^2^ = 9.6			
	*P* < 0.05		*P* < 0.05		*P* < 0.05			
	Heterozygous	Biallelic	Heterozygous	Biallelic	Heterozygous	Biallelic		
	Chi^2^ = 5.1 *P* < 0.05	Chi^2^ = 8.7 *P* < 0.05	Chi^2^ = 6.0 *P* < 0.05	Chi^2^ = 9.7 *P* < 0.05	Chi^2^ = 6.0 *P* < 0.05	Chi^2^ = 5.2 *P* < 0.05		
MDS‐UPDRS part III: Kruskal‐Wallis								
	Combined *GBA1* ^*+*^		Combined *GBA1* ^*+*^		Combined *GBA1* ^*+*^			
	Chi^2^ = 12.4		Chi^2^ = 14.4		Chi^2^ = 9.7			
	*P* < 0.05		*P* < 0.05		*P* < 0.05			
	Heterozygous	Biallelic	Heterozygous	Biallelic	Heterozygous	Biallelic		
	Chi^2^ = 7.3 *P* < 0.05	Chi^2^ = 6.7 *P* < 0.05	Chi^2^ = 17.8 *P* < 0.05	Chi^2^ = 7.2 *P* < 0.05	Chi^2^ = 5.8 *P* < 0.05	Chi^2^ = 9.7 *P* < 0.05		
Serum alpha synuclein: logistic regression							p < 0.05	
					Not significant			

At baseline there were 117 participants (35 controls, 39 heterozygous, and 43 biallelic), 103 at time point 1 (28 controls, 33 heterozygous, and 42 biallelic), and 85 at time point 2 (19 controls, 26 heterozygous, and 40 biallelic); 110 participants (32 controls, 36 heterozygous, and 42 biallelic).

Time point 1 = 2 years; time point 2 = 4 years.

**Figure 2:**
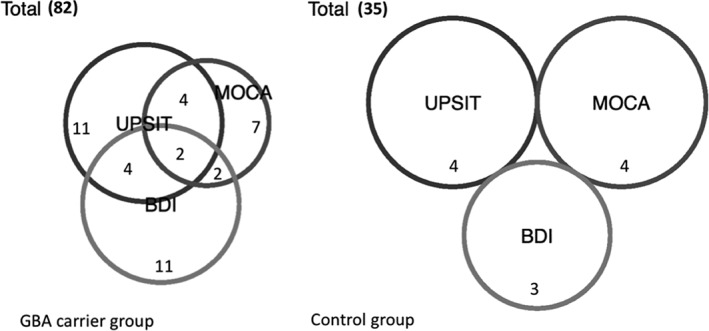
Shows that overlap of participants with the worst/fastest deteriorating BDI, MOCA and UPSIT scores based on our risk stratification system which used as threshold for each test the (approximately) worst 10% of the cohort score for that variable (UPSIT = 3/8: 86th centile and above, MOCA = 3/8: 91th centile and above, BDI =4/8: 86th centile and above) compared with the control group (UPSIT 2/8: 89th centile, MOCA 3/8: 89th centile, BDI: 2/8 87th centile). Subset analysis showed that this clustering effect was present within the bi‐allelic but not the heterozygous group. At baseline there were 117 participants (35 controls, 39 heterozygous and 43 bi‐allelic), 103 at time‐point 1 (28 controls, 33 heterozygous and 42 biallelic) and 85 at time‐point 2 (19 controls, 26 heterozygous and 40 bi‐allelic).

Cognitive impairment was worse at all time points among the combined group of *GBA1* carriers. Among biallelic and heterozygous groups, they were worse at baseline and at time point 2 for the biallelic group (Table 2; Fig. 1). Progression to mild cognitive impairment was worse among the combined *GBA1* group (OR, 4.2; 95% CI: 1.1–16.6; *P* < 0.05), heterozygous (OR, 2.6; 95% CI: 1.6–11.4; *P* < 0.05), and biallelic group (OR, 8.2; 95% CI: 1.6–41.0; *P* < 0.05; Fig.1).

Rates of depressive features were worse at time point 2 only, which was mirrored in subset analysis in the heterozygous, but not the biallelic, group (Table 2). Progression to depressive features was not significantly higher among the *GBA1* group.

MDS‐UPDRS II and III was worse at all time points in the combined *GBA1* group, the heterozygous group, and the biallelic group at all time points (Table 2; Fig. 1).

There were no significant differences cross‐sectionally or prospectively for RBDSQ scores (Table 2). There was no association between the number of severe mutations and any clinical markers at any time point either within the combined GBA, biallelic, or heterozygous subgroups. For prospective analyses, we confirmed our findings represented a true change in score across the *GBA1* group using a Cuzick trend analysis (olfaction Z = –19.17, *P* < 0.05; cognition Z = –3.72, *P* < 0.05).

### Serum A‐SYN Is Increased in High‐Risk GBA1 Mutation Carriers

Serum A‐SYN was higher in those with higher combined risk scores (UPSIT + MoCA + BDI: OR, 1.4; 95% CI: 1.0–1.9; *P* < 0.05). Moreover, A‐SYN serum concentration appeared to correlate with the number of “severe” *GBA1* mutations carried (OR, 1.4; 95% CI: 1.05–1.77; *P* < 0.05).

### Illustrative Case GBA1 Biallelic Participant Who Developed PD

At baseline evaluation in March 2010, a 50‐year‐old male with established GD had no clinical features of PD. By March 2013, when he was 53 years old, he had developed bradykinesia, rigidity, and a unilateral resting tremor and a marked cognitive deficit, which fulfilled diagnostic criteria for PD. He was homozygous for R463C, a severe GD‐causing mutation^17^ which has been described in several patients with PD and dementia with Lewy bodies.^1^, ^3^, ^18^ His serum (A‐SYN) concentrations were the highest in the cohort.

### Clustering of Prodromal PD Symptoms Among a Subset of GBA1 Carriers

We hypothesized the existence of a high‐risk group with accelerated progression of a cluster of prodromal features. We devised a new methodology (see Risk Stratification Procedure section and Supporting Information Fig. S1 for details), which took into account the progression and severity of each prodromal feature. We then used a backward‐selection ordinal logistic regression model to establish which, if any, of these features were associated with olfactory deficits, the prodromal feature found by our analysis to be most affected in *GBA1* cases.

This model revealed that those with olfactory disturbance were clustered with impaired cognition (MoCA: OR, 1.5; 95% CI: 0.0–2.8; *P* < 0.05) and depression (BDI: OR, 1.3; 95% CI: 0.6–2.8; *P* < 0.05). Figure 2 shows that overlap of (approximately) the worst 10% of participants with the worst/fastest deteriorating BDI, MoCA, and UPSIT scores based on our risk stratification system (UPSIT = 3/8: 86th centile and above; MoCA = 3/8: 91th centile and above; BDI = 4/8: 86th centile and above) compared to the control group (UPSIT 2/8: 89th centile; MoCA 3/8: 89th centile; BDI 2/8: 87th centile). Subset analysis showed that this clustering also occurred among biallelic *GBA1* carriers alone, in terms of cognition (MoCA: OR, 2.95; 95% CI: 1.7–4.5; *P* = < 0.05), but not depression. There were no correlations between these risk scores among the control group (Fig. 2). The combined risk score was not correlated with the number of severe disease‐causing mutations.

### MDS Prodromal PD Criteria

For a probable diagnosis of prodromal PD by the MDS criteria, an arbitrary cutoff of 80% was applied. At baseline, none of the cohort met these criteria. The subject who developed PD did not reach the diagnostic criteria until the last assessment 4 months after his diagnosis was made. His posttest probability scores prediagnosis were respectively 11% and 64%. A breakdown of his assessment scores together with the posttest probabilities derived from the MDS prodromal PD criteria (and our novel stratification scores) are shown in Supporting Information S3.

## Discussion

Our results confirm our previous finding that on longitudinal analysis, impaired olfaction, cognitive impairment, and the motor prodromal signs and symptoms of idiopathic PD are more pronounced among *GBA1* mutation carriers.^11^ Currently, clinical trials of a number neuroprotective compounds targeting the GBA pathway are ongoing. Within this “at‐risk” group, stratification and identification of those likely to convert to PD is a priority.

RBD is considered a sensitive prodromal feature of PD. Previous studies have shown a higher rate of *GBA1* mutations among polysomnographically confirmed RBD cases.^20^ Our study here did not find such an association at 4 to 5 years, but this probably reflects a limitation of power and, potentially, the sensitivity of the RBDSQ to detect cases.

The finding of raised levels of A‐SYN in the serum of “high‐risk” carriers is noteworthy. Both raised and lowered levels of serum A‐SYN have been described in the context of PD and aging and has not proven a robust marker of PD progression. Nevertheless, it is interesting that the 1 subject who developed PD had the highest levels of A‐SYN in the entire cohort, and that the number of severe *GBA1* mutations appeared to correlate with the concentration of serum A‐SYN.

It is notable that neither cross‐sectionally nor prospectively did any of the prodromal features correlate with the number of severe mutations carried. Our findings suggest that the severity and rate of progression of prodromal PD symptoms is not influenced by the number of severe *GBA1* mutations. This may be a symptom of the logistic regression model we used, which, because of its reliance on thresholds, may miss subtle differences in prodromal PD feature severity.

Our novel risk stratification system identified that olfactory disturbance, cognitive impairment, and depression were clustered together. When this risk stratification system was applied retrospectively to our subject who developed PD, his scores were consistently at the 99th centile of the cohort. In contrast, the MDS prodromal PD diagnostic criteria failed to make a diagnosis of prodromal PD, implying that the MDS criteria lack the sensitivity to detect prodromal PD changes in this population. There is some evidence to suggest a more variable clinical prodrome of *GBA1* PD, which may also explain the poor performance of the MDS criteria.^21^

A recent study in a large cohort of leucine‐rich repeat kinase 2 (LRRK2) to whom the MDS prodromal PD criteria was applied showed 92% diagnostic specificity and 80% sensitivity.^22^ Conversely, in cohort studies in elderly populations, although specificity was very high, sensitivity was estimated at only 14%, 18%, and 54%.^23^, ^24^ It may be that these striking differences are a reflection of variation PD incidence.^23^, ^24^ In RBD^25^ or G2019S LRRK2^26^ enriched populations, PD incidence is substantially higher^5^ and this is reflected in the high likelihood ratios (LRRK2 G2019S = 25, electrophysiologIcally proven RBD = 125) allocated by the MDS criteria. In contrast, *GBA1* mutations score 10 in “severe” and 2 in “mild” mutations. This may explain the apparent low sensitivity of the criteria among the general population (and now in *GBA1* carriers).

### Limitations

The main limitation of the study is the small sample size, which caused a number of problems. Most notably, the low penetrance of *GBA1* mutations means that the study is underpowered to track the natural history of the conversion to PD. It also may be underpowered to detect other PD prodromal features such as RBD. Enrichment of the cohort using more sensitive modalities, such as dopaminergic imaging, polysomnography, or by including only severe mutation carriers, might be helpful.

The high attrition rate is an issue. While we used multiple imputation to adjust for dropouts, we cannot completely rule out the possibility of biases influencing dropout, or conversely the character of the dropouts skewing results. For instance, there was disproportionate dropout of older males who are at a higher risk of phenoconversion. Accordingly, our data may *underestimate* the incidence of PD in the cohort.

MDS‐UPDRS assessments were carried out by different assessors at each time point, and this may have introduced bias; however, we minimized the influence of this by testing for variation in MDS UPDRS scores and found high intra‐assessor concordance. Biallelic participants were younger than controls and heterozygotes, as a result of our recruitment strategy which targeted parents and siblings of “index” biallelic GD. Given that age was included as a covariate in all analyses (apart from the MDS‐UPDRS II and III), this is unlikely to be a major confounder. Moreover, given that the PD phenotype is age dependent, a younger *GBA1* cohort would be expected to have milder prodromal PD features, meaning, if anything, the differences in prodromal PD features are underestimated.

## Conclusion

This study provides further longitudinal evidence of deteriorating olfaction, cognition, and motor signs and symptoms of parkinsonism among *GBA1* carriers without PD. We found a clustering effect of olfaction, cognitive impairment, and depression in a subset of participants. Given the incomplete penetrance of *GBA1* in PD, larger cohorts are required to map accurately the natural history of PD conversion among *GBA1* carriers (http://www.rapsodistudy.com). We suggest that a combination of *GBA1* genotyping and screening of prodromal PD features may aid the identification of those more likely to develop PD, and, if confirmed, this would aid targeting of future neuroprotective drugs in those without PD. Moreover, such a stratified cohort may prove a more cost‐effective means of adequately powering neuroprotective trials in this group^26^.

## Author Roles

(1) Research Project: A. Conception and Design; B. Acquisition of Data; C. Analysis and Interpretation of Data; (2) Manuscript: A. Writing of the First Draft, B. Review and Critique; (3) Other: A. ELISA.

S.M.: 1B, 1C, 2A, 2B

M.B.: 1B, 2B

J.B.: 1C, 2B

A. McNeil: 1B, 2B

C.P.: 1B, 2B

T.C.: 1B, 2B

D.H.: 1B, 1C, 2B

A. Mehta: 1C, 2B

H.Z.: 2B, 3A

A.H.V.S.: 1A, 1B, 1C, 2A, 2B

## Financial Disclosures

Nothing to report.

## Supporting information


**Supplementary Figure S1** Explanation of risk scoring system

**Supplementary Figure S2** Flow diagrams of recruitment and retention of subjects within the study. 110 participants (32 controls, 36 heterozygous and 42 bi‐allelic) were included in the longitudinal analysis.

**Supplementary Figure S3** Trimmed means of (A) Rapid eye movement behavior sleep disorder (RBDSQ) and (B) Beck's depression inventory (BDI) scores of combined cohort of GBA carries (dashed line) and controls (solid line) with exact confidence intervals. At baseline there were 117 participants (35 controls, 39 heterozygous and 43 bi‐allelic), 103 at time‐point 1 (28 controls, 33 heterozygous and 42 bi‐allelic) and 85 at time‐point 2 (19 controls, 26 heterozygous and 40 bi‐allelic)

**Supplementary Table S1:** Table of participant mutations
**Supplementary Table S2:** Trimmed mean + exact confidence intervals) and median scores of prodromal features of PD amongst control, homozygous, bi‐allelic and combined group of *GBA1* carriers. At baseline there were 117 participants (35 controls, 39 heterozygous and 43 bi‐allelic), 103 at time‐point 1 (28 controls, 33 heterozygous and 42 bi‐allelic) and 85 at time‐point 2 (19 controls, 26 heterozygous and 40 bi‐allelic).
**Supplementary Table S3.** Prodromal PD and biomarker features of subject who developed Parkinsonism.
