# Supplementary figures and images for: Evolution and clustering of prodromal parkinsonian features in GBA1 carriers
Source: Mov Disord. 2019 Jun 28;34(9):1365–73. doi: 10.1002/mds.27775 (PMC6790937; doi:10.1002/mds.27775)

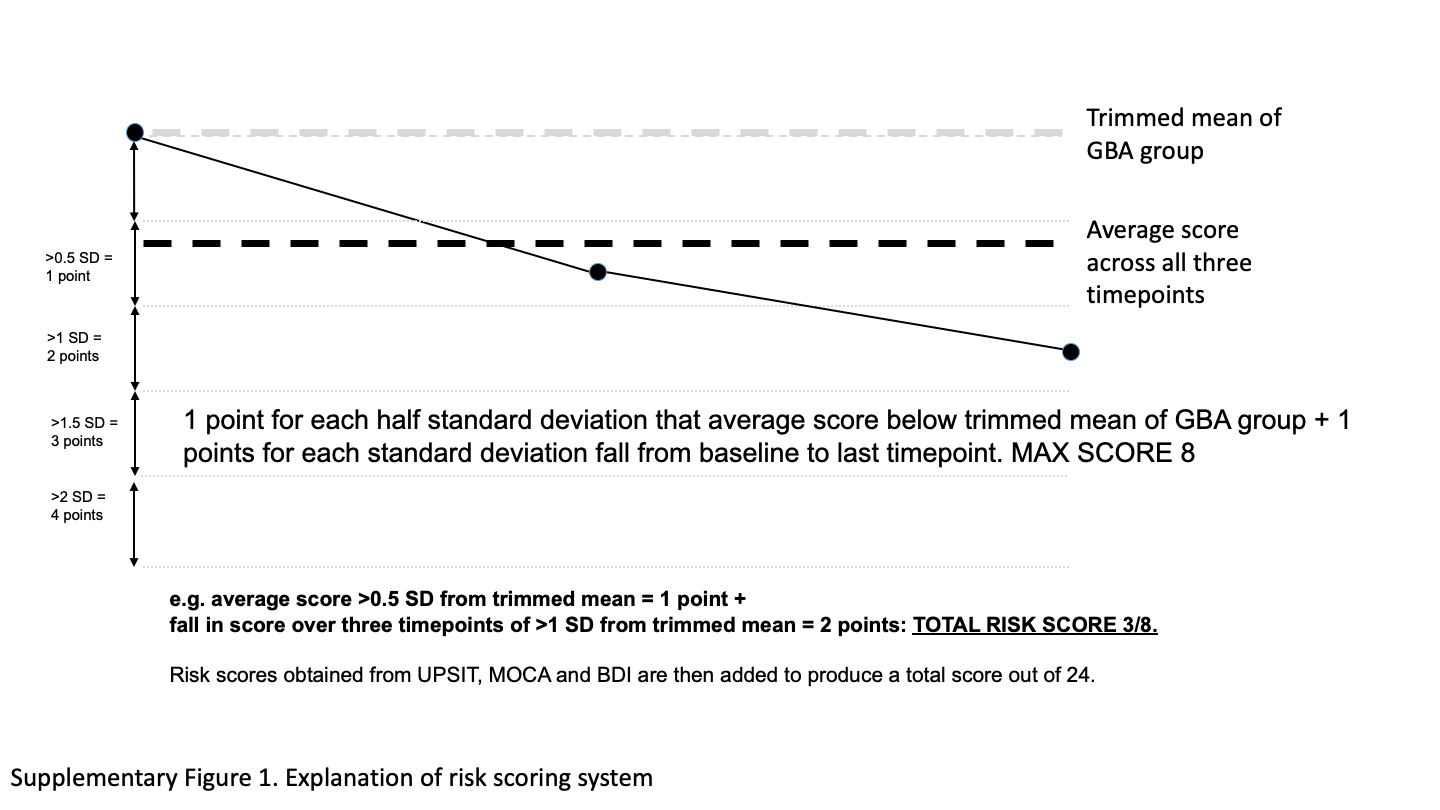

Supplement: Supplementary file 1 — Supplementary Figure S1 Explanation of risk scoring system [file MDS-34-1365-s001.tiff]

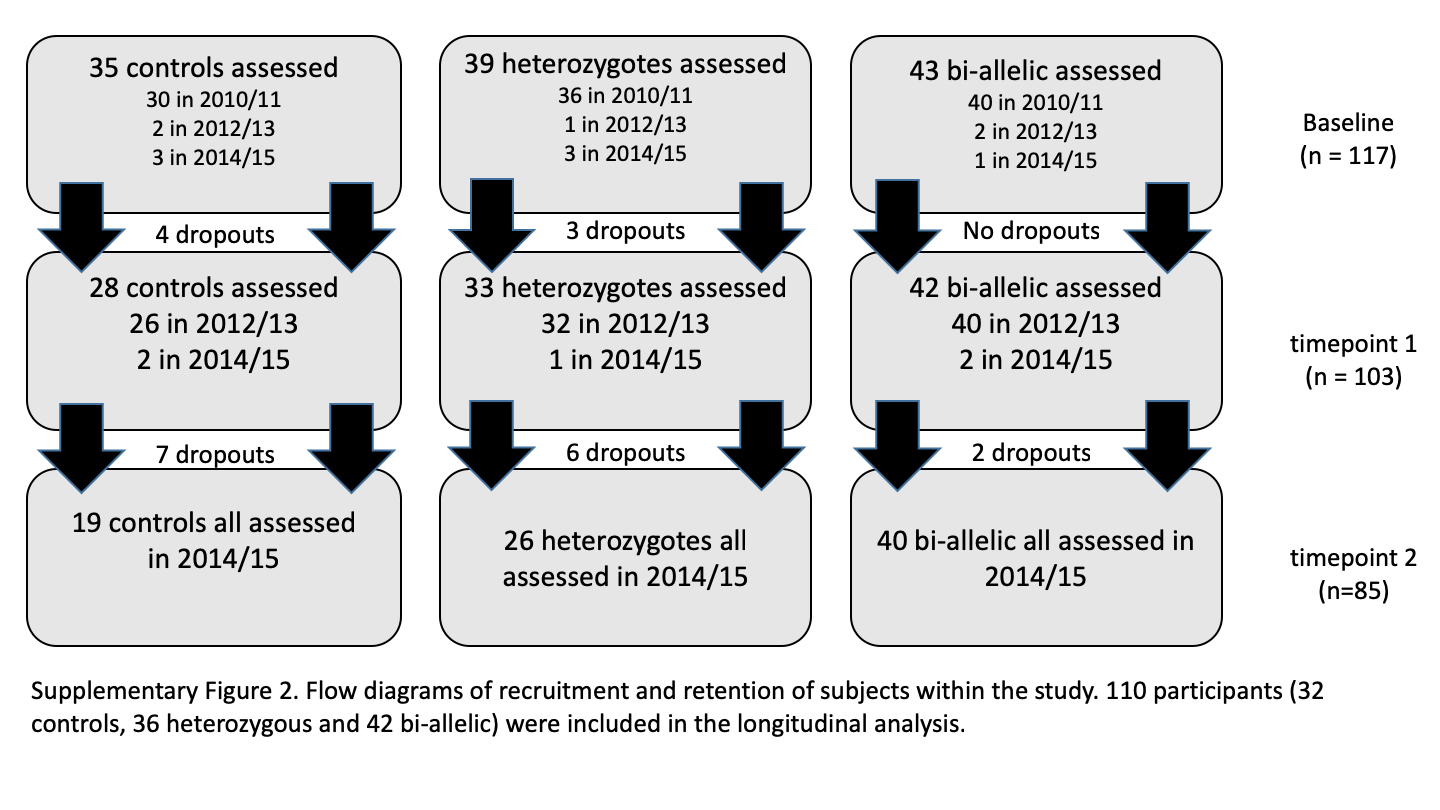

Supplement: Supplementary file 2 — Supplementary Figure S2 Flow diagrams of recruitment and retention of subjects within the study. 110 participants (32 controls, 36 heterozygous and 42 bi‐allelic) were included in the longitudinal analysis. [file MDS-34-1365-s002.tiff]

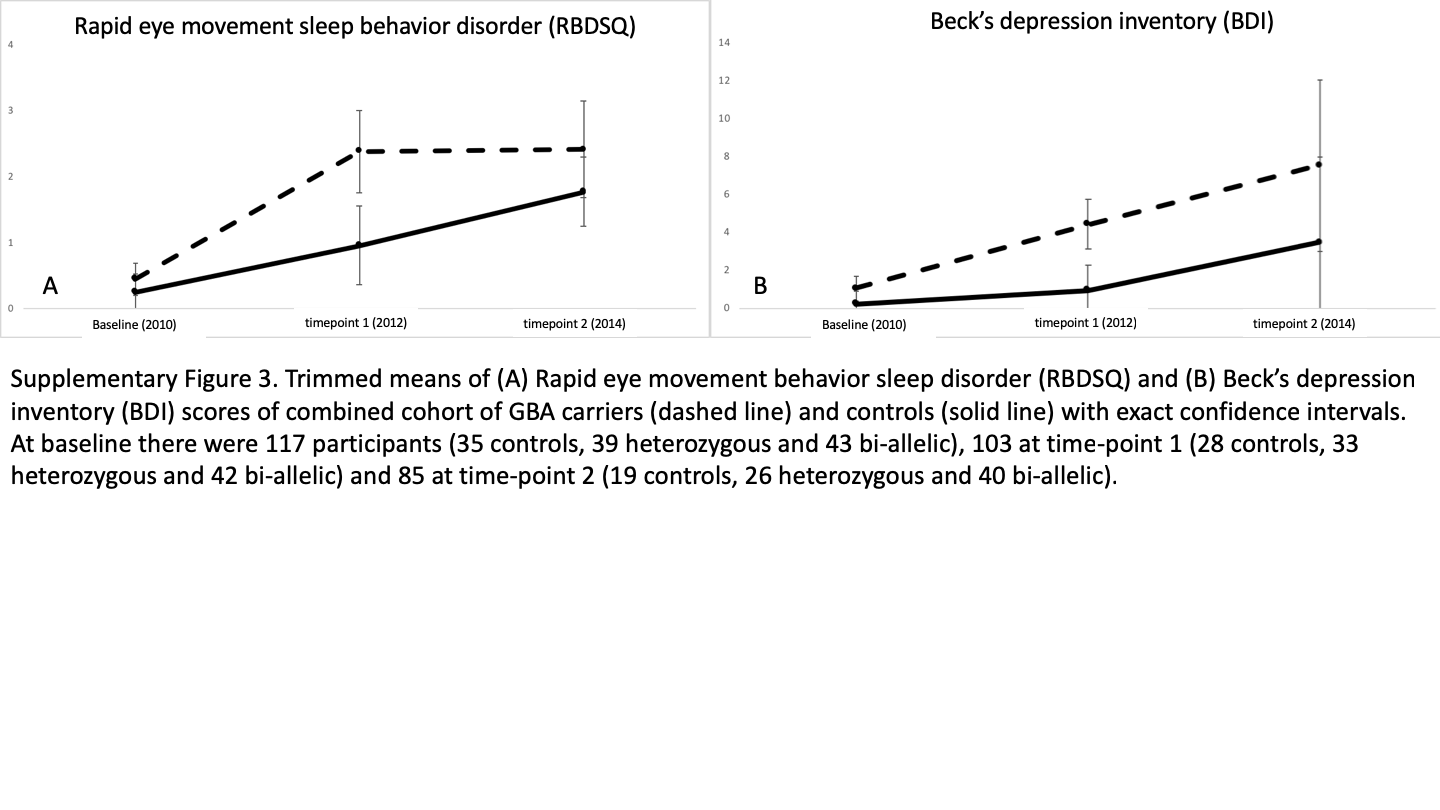

Supplement: Supplementary file 3 — Supplementary Figure S3 Trimmed means of (A) Rapid eye movement behavior sleep disorder (RBDSQ) and (B) Beck's depression inventory (BDI) scores of combined cohort of GBA carries (dashed line) and controls (solid line) with exact confidence intervals. At baseline there were 117 participants (35 controls, 39 heterozygous and 43 bi‐allelic), 103 at time‐point 1 (28 controls, 33 heterozygous and 42 bi‐allelic) and 85 at time‐point 2 (19 controls, 26 heterozygous and 40 bi‐allelic) [file MDS-34-1365-s003.tiff]
