# Supplementary material for: Evolution and clustering of prodromal parkinsonian features in GBA1 carriers
Source: Mov Disord. 2019 Jun 28;34(9):1365–73. doi: 10.1002/mds.27775 (PMC6790937; doi:10.1002/mds.27775)
Supplement: Supplementary file 4 — Supplementary Table S1: Table of participant mutations Supplementary Table S2: Trimmed mean + exact confidence intervals) and median scores of prodromal features of PD amongst control, homozygous, bi‐allelic and combined group of GBA1 carriers. At baseline there were 117 participants (35 controls, 39 heterozygous and 43 bi‐allelic), 103 at time‐point 1 (28 controls, 33 heterozygous and 42 bi‐allelic) and 85 at time‐point 2 (19 controls, 26 heterozygous and 40 bi‐allelic). Supplementary Table S3. Prodromal PD and biomarker features of subject who developed Parkinsonism. [file MDS-34-1365-s004.docx]

| **Supplementary table 1 – Table of participant mutations** | | | | |
| --- | --- | --- | --- | --- |
| **First mutation** | **Severe?** | **Second mutation** | **Severe?** | **Number of genotype** |
| N370S c.1226A>G p.Asn409Ser | N | - | - | 20 |
| N370S c.1226A>G p.Asn409Ser | N | L444P c.1448T>C p.Leu483Pro | Y | 10 |
| N370S c.1226A>G p.Asn409Ser | N | N370S c.1226A>G p.Asn409Ser | N | 7 |
| L444P c.1448T>C p.Leu483Pro | Y | - | - | 5 |
| R463C c.1504C>T p.Arg502Cys | Y | R463C c.1504C>T p.Arg502Cys | Y | 4 |
| W184R c.667T>C p.Trp223Arg | N | - | - | 3 |
| R463C c.1504C>T p.Arg502Cys | Y | - | - | 2 |
| V394L c.1297G>T p.Val433Leu | Y | - | - | 2 |
| N370S c.1226A>G p.Asn409Ser | N | RecNcil, c.1448T>C c.1483G>C c.1497G>C, L444P A456P V460 | Y | 2 |
| N370S c.1226A>G p.Asn409Ser | N | 84GG c.84dupG p.Leu29AlafsX18 | Y | 2 |
| F216Y c.764T>A p.Phe255Tyr | N | - | - | 1 |
| G250V c.856G>T p.Gly289Val | N | - | - | 1 |
| R496H c.1604G>A p.Arg535His | N | - | - | 1 |
| T369M c.1223C>T p.Thr408Met | N | - | - | 1 |
| D409H c.1342G>C p.Asp448His | Y | - | - | 1 |
| RecNcil, c.1448T>C c.1483G>C c.1497G>C, L444P A456P V460 | Y | - | - | 1 |
| N370S c.1226A>G p.Asn409Ser | N | IVS2+1G>A c.115+1G>A | Y | 1 |
| N370S c.1226A>G p.Asn409Ser | N | IVS9 + 1G > A c.1388+1 G>A | N | 1 |
| N370S c.1226A>G p.Asn409Ser | N | V447E c.1457T > A Val486Glu | N | 1 |
| N370S c.1226A>G p.Asn409Ser | N | R120W c.475C>T p.Arg159Trp | N | 1 |
| N370S c.1226A>G p.Asn409Ser | N | L66P c.314T>C p.Leu105Pro | N | 1 |
| N370S c.1226A>G p.Asn409Ser | N | G250V c.856G>T p.Gly289Val | N | 1 |
| N370S c.1226A>G p.Asn409Ser | N | L105R c.431T>G p.Leu144Arg | N | 1 |
| N370S c.1226A>G p.Asn409Ser | N | c1263del55 c.1263-1317del | Y | 1 |
| N370S c.1226A>G p.Asn409Ser | N | D409H c.1342G>C p.Asp448His | Y | 1 |
| N370S c.1226A>G p.Asn409Ser | N | R359X c.1192C>T p.Arg398X | Y | 1 |
| N370S c.1226A>G p.Asn409Ser | N | R463C c.1504C>T p.Arg502Cys | Y | 1 |
| N370S c.1226A>G p.Asn409Ser | N | D380A c.1256A>C p.Asp419Ala | Y | 1 |
| L444P c.1448T>C p.Leu483Pro | Y | F216Y c.764T>A p.Phe255Tyr | N | 1 |
| RecNcil, c.1448T>C c.1483G>C c.1497G>C, L444P A456P V460 | Y | R262G c.901C > G Arg301Gly + S271G | N | 1 |
| L444P c.1448T>C p.Leu483Pro | Y | 595-596delCT c.595_596delCT p.Leu199AspfsX62 | N | 1 |
| R496H c.1604G>A p.Arg535His | Y | R359X c.1192C>T p.Arg398X | Y | 1 |
| D409H c.1342G>C p.Asp448His | Y | D409H c.1342G>C p.Asp448His | Y | 1 |
| L444P c.1448T>C p.Leu483Pro | Y | c.46A>G S16G, IVS2+1 G>A | Y | 1 |

| **Supplementary Table 2. Trimmed mean + exact confidence intervals) and median scores of prodromal features of PD amongst control, homozygous, bi-allelic and combined group of *GBA1* carriers. At baseline there were 117 participants (35 controls, 39 heterozygous and 43 bi-allelic), 103 at time-point 1 (28 controls, 33 heterozygous and 42 bi-allelic) and 85 at time-point 2 (19 controls, 26 heterozygous and 40 bi-allelic).** | | | | | | | | | |
| --- | --- | --- | --- | --- | --- | --- | --- | --- | --- |
|  |  | **baseline** | **time-point 1** | **time-point 2** |  |  | **baseline** | **time-point 1** | **time-point 2** |
| **UPSIT control** | **trimmed mean [95%CI]** | **34.6 [33.6-35.7]** | **34.2 [32.9-35.5]** | **33.1 [31.6-34.6]** | **MDS UPDRS II control** | **trimmed mean [95%CI]** | **0.0** | **0.4 [0.1-0.7]** | **0.7 [0.0-1.3]** |
|  | **median** | 35.0 | 35.0 | 32.0 |  | **median** | 0.0 | 0.0 | 0.0 |
| **UPSIT bi-allelic** | **trimmed mean [95%CI]** | **32.8 [31.4-34.2]** | **31.2 [29.3-33.0]** | **29.8 [2.7-31.9]** | **MDS UPDRS II bi-allelic** | **trimmed mean [95%CI]** | **0.6 [0.1-1.2]** | **2.1 [1.1-3.0]** | **4.8 [2.0-7.5]** |
|  | **median** | 34.0 | 32.0 | 32.0 |  | **median** | 0.0 | 1.0 | 3.0 |
| **UPSIT heterozygous** | **trimmed mean [95%CI]** | **31.6 [29.8-33.4]** | **30.6 [29.0-32.2]** | **29.1 [27.0-31.2]** | **MDS UPDRS II heterozygous** | **trimmed mean [95%CI]** | **0.5 [0.13-0.8]** | **1.3 [0.8-1.7]** | **2.1 [1.0-3.2]** |
|  | **median** | 33.5 | 32.0 | 30.0 |  | **median** | 0.0 | 1.0 | 1.0 |
| **UPSIT gba** | **trimmed mean [95%CI]** | **32.2 [31.1-33.5]** | **31.1 [30.1-32.1]** | **29.7 [28.2-31.2]** | **MDS UPDRS II gba** | **trimmed mean [95%CI]** | **0.5 (0.1-0.9)** | **1.6 [1.0-2.1]** | **3.3 [1.8-4.8]** |
|  | **median** | 34.0 | 32.0 | 30.0 |  | **median** | 0.0 | 1.0 | 2.0 |
| **MoCA control** | **trimmed mean [95%CI]** | **27.9 [27.3-28.4]** | **28.0 [27.5-28.7]** | **28.3 [27.7-29.0]** | **MDS UPDRS III control** | **trimmed mean [95%CI]** | **0.0 [0.0-0.2]** | **1.9 [0.4-3.3]** | **2.5 [1.0-4.0]** |
|  | **median** | 28.0 | 28.0 | 28.5 |  | **median** | 0.0 | 0.0 | 1.0 |
| **MoCA bi-allelic** | **trimmed mean [95%CI]** | **26.6 [25.8-27.4]** | **26.9 [25.8-28.0]** | **25.8 [24.5-27.0]** | **MDS UPDRS III bi-allelic** | **trimmed mean [95%CI]** | **1.9 [0.2-3.6]** | **6.4 [3.0-9.7]** | **8.5 [4.8-12.2]** |
|  | **median** | 27.0 | 28.5 | 26.0 |  | **median** | 0.0 | 3.0 | 5.0 |
| **MoCA heterozygous** | **trimmed mean [95%CI]** | **26.1 [25.1-27.1]** | **26.5 [25.4-27.6]** | **26.3 [24.9-27.7]** | **MDS UPDRS III heterozygous** | **trimmed mean [95%CI]** | **2.4 [0.9-3.8]** | **8.0 [5.4-10.6]** | **6.5 [3.7-9.2]** |
|  | **median** | 27.0 | 27.0 | 26.5 |  | **median** | 0.0 | 8.0 | 7.0 |
| **MoCA gba** | **trimmed mean [95%CI]** | **26.4 [25.8-27.1]** | **26.8 [26.0-27.5]** | **26.0 [25.1-26.9]** | **MDS UPDRS III gba** | **trimmed mean [95%CI]** | **1.9 [0.9 - 3.0]** | **6.8 [4.7-8.9]** | **7.6 [5.2-9.9]** |
|  | **median** | 27.0 | 27.0 | 26.0 |  | **median** | 0.0 | 4.0 | 5.0 |
| **BDI control** | **trimmed mean [95%CI]** | **0.72 [0.0-1.77]** | **2.1 [0.0-5.0]** | **3.44 [1.8-5.1]** | **RBDQS control** | **trimmed mean [95%CI]** | **0.1 [0.0-0.3]** | **1.0 [0.3-1.6]** | **1.8 [1.1-2.5]** |
|  | **median** | 0.0 | 0.0 | 3.5 |  | **median** | 0.0 | 0.0 | 2.0 |
| **BDI bi-allelic** | **trimmed mean [95%CI]** | **1.24 [0.3-2.2]** | **5.3 [3.1-7.5]** | **9.3 [6.5-12.0]** | **RBDSQ bi-allelic** | **trimmed mean [95%CI]** | **0.6 [0.1-1.2]** | **2.8 [1.9-3.6]** | **2.5 [1.8-3.1]** |
|  | **median** | 0.0 | 0.0 | 8.0 |  | **median** | 0.0 | 2.0 | 2.0 |
| **BDI heterozygous** | **trimmed mean [95%CI]** | **1.41 [0.1-2.7]** | **3.8 [2.5-5.1]** | **6.0 [4.5-7.4]** | **RBDSQ heterozygous** | **trimmed mean [95%CI]** | **0.6 [0.1-1.0]** | **2.1 [1.2-3.0]** | **2.4 [1.6 3.1]** |
|  | **median** | 0.0 | 3.0 | 5.0 |  | **median** | 0.0 | 1.5 | 2.5 |
| **BDI gba** | **trimmed mean [95%CI]** | **1.3 [0.5-2.0]** | **4.4 [3.0-5.7]** | **7.5 [5.7-9.3]** | **RBDSQ gba** | **trimmed mean [95%CI]** | **0.5 [0.2-0.9]** | **2.4 [1.8-3.0]** | **2.4 [1.9-2.9]** |
|  | **median** | 0.0 | 4.0 | 7.5 |  | **median** | 0.0 | 2.0 | 2.0 |
| ***University of Pennsylvania smell identification test, MoCA: Montreal cognitive assessment, BDI: Beck’s depression index, RBDSQ: Rapid eye movement sleep behavior disorder questionnaire. UMSARS: Unified multiple system atrophy ratings scale, MDS UPDRS: Movement disorders society Unified Parkinson disease rating scale*** | | | | | | | | | |

| **Supplementary Table 3. Prodromal PD and biomarker levels features of subject who developed Parkinsonism.** | | | |
| --- | --- | --- | --- |
|  | Baseline (2010)  Score [centile] | Time-point 1 (2012)  Score [centile] | Time-point 2 (2014)  Score [centile]  *Parkinsonism DIAGNOSIS |
| UPSIT | 20 [93] | 14 [96] | 17 [95] |
| MoCA | 27 [54] | 27 [57] | 21 [87] |
| BDI | 4 [82] | 4 [50] | 25 [95] |
| RBDSQ | 0 | 2 [52] | 5 [85] |
| MDS UPDRS II | 0 | 3 [85] | 29 [99] |
| MDS UPDRS III | 0 | 3 [50] | 45 [99] |
| Serum alpha synuclein | - | - | [100] |
| Post test probability by MDS criteria | 11% | 64% | 91% |
| RISK score | 8/12 [99] | 12/24 [99] | 20/24 [99] |
| *University of Pennsylvania smell identification test, MoCA: Montreal cognitive assessment, BDI: Beck’s depression index, RBDSQ: Rapid eye movement sleep behavior disorder questionnaire. UMSARS: Unified multiple system atrophy ratings scale, MDS UPDRS: Movement disorders society Unified Parkinson disease rating scale* | | | |
